# Developmental and Socioeconomic Gradients in Perceived Mental Health and Mood Disorder Risk Among Children and Adolescents: A Population-Based Parent-Report Study

**DOI:** 10.3390/healthcare14060763

**Published:** 2026-03-18

**Authors:** Karolina Klimek, Teresa Wagner-Tomaszewska, Tomasz Jurys, Zofia Spandel, Mateusz Grajek

**Affiliations:** 1 Department of Economics and Health Care Management, Faculty of Public Health in Bytom, Medical University of Silesia in Katowice, 40-007 Katowice, Poland; karolina.klimek@sum.edu.pl; 2 Department of Public Health, Faculty of Public Health in Bytom, Medical University of Silesia in Katowice, 41-902 Bytom, Polandzofiaspandel2001@gmail.com (Z.S.); 3 Department of Rehabilitation, Faculty of Health Sciences in Katowice, Medical University of Silesia in Katowice, 41-902 Bytom, Poland

**Keywords:** child and adolescent mental health, socioeconomic determinants, mood disorder risk, developmental gradient, parental assessment

## Abstract

**Background**: Mental health problems in childhood and adolescence constitute a major public health concern, influencing developmental trajectories, educational outcomes, and long-term well-being. This study aimed to assess developmental and socioeconomic gradients in perceived mental health and mood disorder risk among children and adolescents, integrating parental evaluations, symptom-related indicators, and sociodemographic correlates. **Methods**: A cross-sectional survey was conducted among 1177 parents or legal guardians of children aged 6–18 years in Poland. Data were collected via a structured questionnaire assessing perceived physical and mental health, socioeconomic characteristics, and seven symptom-based items aligned with the Children’s Depression Inventory 2 (CDI-2) diagnostic framework. Nonparametric tests (χ^2^, Spearman’s ρ, Kruskal–Wallis H) were applied to examine age-related differences and socioeconomic gradients in perceived mental health and mood disorder risk. **Results**: Parental evaluations indicated a consistent discrepancy between physical and mental health, with psychological well-being rated less favorably and exhibiting greater variability. Both perceived mental health and mood disorder risk showed strong age-related differentiation, revealing declining scores with increasing age (ρ < 0, *p* < 0.001). Family financial situation demonstrated the strongest association with mental health outcomes (H = 71.39, *p* < 0.001), while parental occupational status exerted moderate effects and educational attainment showed no significant influence. Concentration difficulties, affective distress, and somatic symptoms such as fatigue and sleep disturbance were commonly reported. **Conclusions**: Findings indicate that child and adolescent mental health is shaped by interacting developmental and socioeconomic determinants. Adolescence and financial disadvantage represent key vulnerability factors associated with poorer psychological outcomes. The results highlight the need for developmentally targeted and socially equitable mental health strategies within pediatric and preventive healthcare systems.

## 1. Introduction

Mental health in childhood and adolescence has emerged as a critical public health priority, reflecting its fundamental role in shaping developmental trajectories, educational attainment, social functioning, and long-term health outcomes. Contemporary models of health increasingly emphasize the inseparability of psychological and somatic well-being, positioning mental health as a core component of overall health rather than a secondary or derivative domain. The World Health Organization (WHO) underscores that mental disorders constitute one of the leading causes of disability among young people globally, with early onset frequently extending into adulthood and contributing to cumulative functional impairment [1]. Mental health in childhood and adolescence should be interpreted within a developmental framework, as emotional regulation, cognitive functioning, and social competencies undergo substantial changes across successive stages of development. Developmental transitions—particularly the shift from late childhood to adolescence—are associated with increasing psychosocial demands, identity formation processes, and heightened sensitivity to environmental stressors. These mechanisms contribute to age-related variability in mental health outcomes and may partly explain the increased vulnerability to internalizing symptoms observed during adolescence [2].

Epidemiological evidence accumulated over recent years indicates a substantial and, in many contexts, increasing prevalence of mental health difficulties among children and adolescents. Large-scale global analyses estimate that approximately one in seven individuals aged 10–19 years experiences a diagnosable mental disorder, with anxiety and depressive disorders representing the most common categories [1]. Notably, recent meta-analytic data have documented marked increases in the prevalence of depressive and anxiety symptoms during and following the COVID-19 pandemic [2]. Santomauro et al. demonstrated a significant global rise in both major depressive disorder and anxiety disorders, disproportionately affecting younger populations [3]. Complementary findings by Racine et al. revealed that clinically elevated symptoms of depression and anxiety among children and adolescents nearly doubled compared to pre-pandemic estimates [4]. These trends highlight the heightened vulnerability of younger cohorts to environmental stressors and systemic disruptions.

Developmental psychopathology frameworks provide a theoretical lens for interpreting age-related variability in mental health outcomes. Within the developmental psychopathology perspective, mental health outcomes are understood as the result of dynamic interactions between biological maturation, psychological processes, and environmental influences across the life course. This framework emphasizes that risk and protective factors accumulate over time and that developmental transitions may either amplify vulnerability or promote resilience depending on contextual conditions. Childhood and adolescence are characterized by dynamic neurobiological maturation, evolving cognitive capacities, identity formation processes, and intensifying social demands [5]. Adolescence, in particular, represents a developmental period associated with increased risk for affective disorders, emotional dysregulation, and internalizing symptomatology [6]. Neurodevelopmental changes affecting reward sensitivity, stress reactivity, and executive functioning are believed to interact with psychosocial stressors, thereby amplifying susceptibility to mood disturbances [6]. Empirical studies consistently demonstrate age gradients in the prevalence of depressive symptoms, with incidence rates increasing during early and mid-adolescence [4].

Beyond developmental determinants, a substantial body of literature has documented the role of socioeconomic conditions as key predictors of child and adolescent mental health. A growing body of epidemiological research indicates a rising prevalence of emotional difficulties and mood-related symptoms among children and adolescents in many countries. Large population-based studies have documented increases in depressive symptoms, anxiety, and psychological distress, particularly during early and mid-adolescence, highlighting the need to better understand developmental and socioeconomic determinants of youth mental health. Socioeconomic adversity has been associated with elevated risk of emotional and behavioral disorders, poorer psychological well-being, and increased exposure to chronic stressors. Mechanistic explanations emphasize pathways involving financial strain, parental stress, reduced access to resources, neighborhood disadvantage, and diminished opportunities for protective psychosocial experiences [7]. Recent analyses confirm the persistence of a socioeconomic gradient in mental health outcomes, whereby children from economically disadvantaged families exhibit disproportionately higher levels of psychological distress [7]. The WHO explicitly identifies social and economic inequalities as central structural determinants contributing to disparities in mental health [1].

Family context represents an especially salient environment influencing psychological development. Parental employment status, financial stability, and psychosocial functioning are recognized as factors exerting both direct and indirect effects on child mental health. Economic insecurity and employment instability may increase familial stress exposure, disrupt caregiving processes, and constrain access to supportive interventions [7]. Conversely, stable occupational engagement may function as a protective factor through enhanced predictability, social integration, and resource availability [8]. Parental assessments represent an important observational perspective on children’s mental health, particularly in population-based research where large-scale clinical assessment is not feasible. At the same time, parental reports reflect a specific interpretative context shaped by family environment, parental stress, and expectations regarding children’s functioning. Consequently, parental evaluations should be understood as indicators of perceived psychological well-being rather than direct clinical assessments [7,8].

Parallel to the epidemiological burden, concerns regarding accessibility and adequacy of mental health services for children and adolescents have intensified. International reports consistently document gaps in service provision, long waiting times, workforce shortages, and systemic barriers limiting effective utilization of care [1,9]. The Organisation for Economic Co-operation and Development (OECD) highlights that unmet mental health needs among young people remain a widespread challenge across high-income countries, with early detection and preventive services frequently underdeveloped relative to demand [9]. These structural limitations may contribute to delayed intervention, exacerbation of symptoms, and the widening of health inequalities.

Parental perceptions constitute an important yet comparatively underexplored dimension within the mental health research landscape. Parents function as primary observers, gatekeepers of care, and decision-makers regarding help-seeking behaviors. Their evaluations of child well-being, recognition of symptoms, and appraisal of service accessibility significantly influence pathways to diagnosis and treatment [10]. Discrepancies between subjective global assessments and symptom-specific indicators have been documented, suggesting that perceived mental health status may not fully capture subclinical or emerging difficulties [11].

In light of the growing prevalence of mental health difficulties, the established role of developmental and socioeconomic determinants, and persistent concerns regarding service accessibility, the present study seeks to contribute to the empirical understanding of perceived mental health needs among children and adolescents. By integrating parental evaluations, symptom-related risk indicators, and sociodemographic correlates, the study addresses a critical interface between epidemiological patterns and real-world health system challenges.

The present study was designed to conduct a multidimensional assessment of perceived physical and mental health status among children and adolescents aged 6–18 years, as evaluated by their parents or legal guardians. The investigation pursued three analytically interrelated objectives. First, it sought to quantify the distribution of parental evaluations of children’s physical and mental health and determine whether systematic age-related gradients could be observed. Second, it aimed to operationalize and estimate the level of risk for mood disorders based on symptom-related indicators reflecting affective, cognitive, and somatic functioning. Third, the study examined the associations between mental health indicators and selected sociodemographic variables, including child age, parental educational attainment, parental occupational status, and family financial situation.

The conceptual framework underlying the study assumed that mental health outcomes in childhood and adolescence are shaped by interacting developmental and environmental determinants. Consequently, specific hypotheses were formulated: (1) parental assessment of mental health would demonstrate a negative association with child age, (2) socioeconomic variables—particularly perceived family financial status—would significantly differentiate parental evaluations of children’s mental health and the risk of mood disorder symptoms, with less favorable financial conditions expected to be associated with poorer perceived mental health and higher symptom burden, (3) parental occupational activity would exhibit measurable associations with child mental health outcomes, and (4) parental educational attainment would not constitute a primary differentiating factor. These hypotheses were evaluated using inferential statistical procedures applied to cross-sectional survey data.

## 2. Materials and Methods

The study employed a cross-sectional survey design grounded in the diagnostic poll methodology. Data were collected using a structured questionnaire administered to 1177 parents or legal guardians, each providing information concerning one child. The analytic sample thus comprised 1177 children aged 6–18 years (mean age = 11.9 years; median = 12 years). To enable developmentally sensitive comparisons, respondents were stratified into three age cohorts: early childhood (6–9 years), middle childhood (10–14 years), and adolescence (15–18 years).

Participants were recruited through a population-based survey targeting parents or legal guardians of children aged 6–18 years residing in Poland. Data were collected using a structured questionnaire distributed online through educational institutions and parent networks. Participation was voluntary and anonymous, and respondents provided information regarding one child within the specified age range.

Health status assessments were obtained via ordinal scales encompassing five response categories, ranging from “very poor” to “very good.” This measurement approach justified the use of nonparametric statistical techniques. Associations between categorical variables were examined using Pearson’s chi-square (χ^2^) test, while effect sizes were estimated with Cramér’s V coefficient. Relationships involving ordinal variables were evaluated using Spearman’s rank-order correlation (ρ), given its robustness under non-normal distributional assumptions. Differences in continuous or quasi-continuous indicators (e.g., aggregated mood disorder risk index) across multiple groups were assessed using the Kruskal–Wallis H test, followed by post hoc pairwise comparisons where appropriate.

Risk of mood disorders was operationalized through a composite index derived from seven symptom-oriented items conceptually aligned with the CDI-2 diagnostic framework. Each item captured frequency-based manifestations of psychological distress observed during the preceding four weeks. Responses were reverse-scored, 1 = highest symptom frequency, 5 = absence of symptoms, and the arithmetic mean across items constituted the global risk indicator. Lower values therefore denoted higher risk. Internal consistency was assumed to be acceptable due to the conceptual coherence of items reflecting mood, cognition, sleep, fatigue, and social withdrawal domains. The composite indicator used in this study should be interpreted as a symptom-based index reflecting the presence of mood-related psychological difficulties rather than as a diagnostic estimate of mood disorder risk. The items capture commonly reported emotional, cognitive, and somatic manifestations associated with internalizing symptomatology in youth.

Internal consistency of the seven-item symptom index was assessed using Cronbach’s alpha (0.82). The obtained coefficient indicated acceptable internal coherence of the measure, supporting its use as a composite indicator of mood-related symptomatology in population-level analyses.

The statistical strategy was designed to examine developmental gradients and socioeconomic differentiation in perceived mental health indicators. Given the ordinal character of several variables and the non-normal distribution of selected measures, nonparametric statistical procedures were applied. Age-related differences were examined using the Kruskal–Wallis H test, associations between ordinal variables were assessed using Spearman’s rank correlation coefficient, and categorical distributions were analyzed using χ^2^ tests.

The use of nonparametric statistical procedures reflects an analytic approach appropriate for ordinal data and non-normal distributions, allowing for robust examination of associations and group differences within the structure of the collected variables.

## 3. Results

Parental evaluations indicated a marked discrepancy between perceived physical and mental health status. While physical health was predominantly assessed as “good” or “very good,” mental health ratings exhibited substantially greater dispersion and a higher prevalence of intermediate and negative evaluations. This divergence suggests that psychological functioning is perceived as more vulnerable and variable relative to somatic health.

Age emerged as a central differentiating factor. The distribution of mental health evaluations varied significantly across developmental cohorts (χ^2^ test, *p* < 0.001), revealing a pronounced decline in positive assessments with increasing age. Spearman’s correlation confirmed a statistically significant negative association between child age and mental health evaluation (ρ < 0, *p* < 0.001). The magnitude of this relationship indicates that developmental stage constitutes a nontrivial determinant of perceived psychological well-being (Table 1).

In contrast, parental educational attainment did not significantly differentiate mental health ratings (*p* > 0.05). The absence of association suggests that parental perceptions of child mental health may be relatively independent of educational capital, or that education alone does not sufficiently capture relevant psychosocial resources.

Parental occupational status demonstrated statistically significant associations with both physical and mental health evaluations (*p* < 0.05). Children of economically active parents were more frequently rated as exhibiting favorable mental health. Although effect sizes were modest, the directionality of findings aligns with theoretical models linking occupational stability with reduced familial stress exposure and enhanced psychosocial support structures.

Family financial situation exhibited the strongest associations across analyses. Differences in mental health evaluations between financial categories reached high levels of statistical significance (χ^2^ test, *p* < 0.001), accompanied by large effect sizes. The proportion of positive mental health assessments among children from financially disadvantaged families was markedly reduced. Spearman’s correlation further indicated a positive relationship between financial status and mental health evaluation (ρ > 0, *p* < 0.001), underscoring the gradient-like nature of socioeconomic influences.

The aggregated mood disorder risk index yielded a mean value of 3.80 (SD = 0.59), reflecting moderate population-level risk. However, significant between-group differences were observed (Kruskal–Wallis test, *p* < 0.001). Adolescents displayed significantly lower index values (indicating higher risk) relative to younger cohorts. Spearman’s correlation corroborated the inverse association between age and mood disorder risk (ρ < 0, *p* < 0.001), suggesting cumulative vulnerability across developmental progression (Table 2 and Table 3).

Financial status exerted a substantial effect on mood disorder risk. Children from families reporting poor financial conditions exhibited significantly elevated risk levels (*p* < 0.001), with effect sizes indicative of strong practical significance. Increased variance within this subgroup further suggests heterogeneity of psychological outcomes under conditions of economic strain.

Symptom-level analyses revealed notable prevalence rates of affective and cognitive difficulties. Concentration problems constituted the most frequently reported difficulty, affecting over half of the sample at clinically relevant frequencies. Indicators associated with depressive cognition (negative self-evaluations), affective dysregulation (sadness), and somatic disturbance (sleep difficulties, fatigue) were observed in substantial proportions. These findings collectively point toward a high prevalence of subclinical psychological distress within the population.

Chronic health problems were reported in over one-quarter of children, with a measurable subset involving mental health conditions. The coexistence of somatic and psychological difficulties may reflect bidirectional interactions consistent with psychosomatic and stress-vulnerability models.

Patterns of service utilization revealed reliance on educational and psychological support mechanisms, alongside substantial privatization of specialized mental health care. Perceived low accessibility of publicly funded psychiatric services may therefore represent a structural factor shaping help-seeking behaviors (Table 4).

Overall, the results demonstrate that child age and family financial situation constitute the most salient correlates of perceived mental health and mood disorder risk. These associations exhibit statistical robustness and theoretical coherence, reinforcing developmental and socioeconomic frameworks of child and adolescent mental health.

## 4. Discussion

The results obtained in the present study are consistent with a substantial body of contemporary international literature indicating that mental health difficulties among children and adolescents represent a significant and developmentally differentiated public health challenge. Although parents predominantly evaluated their children’s overall mental health as good or very good, the simultaneous presence of elevated symptom-level indicators suggests a more complex picture of psychological functioning. The findings should be interpreted as reflecting patterns of perceived psychological difficulties reported by parents rather than clinically verified mental disorders. Nevertheless, such indicators may provide valuable insight into early manifestations of emotional distress and population-level gradients in child and adolescent well-being.

A central finding concerns the pronounced age-related gradient observed across both global mental health assessments and the composite mood disorder risk index. The systematic decline in favorable mental health evaluations with increasing age corresponds closely with recent epidemiological evidence. Santomauro et al. documented a marked global increase in depressive and anxiety disorders during the COVID-19 pandemic, emphasizing that younger populations exhibited disproportionately elevated vulnerability [3]. Complementary meta-analytic findings reported by Racine et al. demonstrated high pooled prevalence rates of clinically elevated depressive and anxiety symptoms among children and adolescents, with prevalence estimates increasing with age [4].

Developmental neuroscience frameworks offer theoretical grounding for these gradients. Fuhrmann et al. characterize adolescence as a sensitive neurodevelopmental period marked by intensified emotional reactivity, ongoing maturation of executive regulatory systems, and heightened responsiveness to environmental stressors [12]. These neurobiological processes, interacting with increasing academic demands, social complexity, and identity-related challenges, provide plausible explanatory mechanisms for the elevated mood disorder risk observed among adolescents in the present study.

The magnitude of socioeconomic differentiation, particularly with respect to family financial situation, represents another key observation. Children from economically disadvantaged families exhibited significantly less favorable mental health evaluations and substantially elevated mood disorder risk. These findings closely align with meta-analytic evidence reported by Peverill et al., who demonstrated that lower socioeconomic status is robustly associated with increased internalizing psychopathology across childhood and adolescence [7]. The authors emphasize cumulative risk pathways involving chronic stress exposure, reduced environmental predictability, and diminished access to protective psychosocial resources.

Similarly, Rakesh et al. reported that socioeconomic disadvantage constitutes a significant predictor of adolescent mental disorders, even after accounting for potential confounders [13]. The persistence of socioeconomic gradients across diverse methodological approaches underscores the structural nature of these associations. Financial strain is widely understood to contribute to sustained activation of stress-response systems, alterations in family dynamics, and reduced access to mental health resources, thereby amplifying vulnerability to affective dysregulation.

Although the associations between parental occupational status and child mental health observed in the present study were modest in magnitude, they remain theoretically coherent. Shahidi et al. highlight that employment instability may exert independent effects on youth mental health via mechanisms involving parental stress, reduced family stability, and perceived insecurity [14]. Conversely, occupational stability may function as a protective factor through enhanced predictability and access to material and social resources.

A particularly noteworthy pattern concerns the divergence between generally favorable global mental health evaluations and the high prevalence of symptom-level indicators. Concentration difficulties, sleep disturbances, affective distress, and negative self-referential cognitions were reported at substantial frequencies. This discrepancy has been extensively discussed in the literature on informant-based assessment [15]. Gentile et al. emphasize that discrepancies between global evaluations and symptom-specific indicators are common, particularly for internalizing symptoms that may not be readily observable [16]. Parents may therefore underestimate or incompletely capture subjective emotional experiences, especially in older children and adolescents.

The prevalence of subclinical symptomatology observed in the present study is consistent with international estimates. Racine et al. reported pooled prevalence rates exceeding 20% for clinically elevated depressive symptoms among youth [4]. Importantly, subthreshold symptoms are not clinically trivial. Longitudinal models indicate that early subclinical psychological distress significantly predicts subsequent onset of diagnosable disorders, functional impairment, and reduced quality of life [1]. Consequently, the elevated frequency of symptom indicators observed here may reflect emerging psychological vulnerability rather than transient developmental variation.

The pronounced age-related gradient observed across global mental health evaluations and the mood disorder risk index may reflect developmental processes characteristic of adolescence. This period is marked by rapid biological maturation, increasing academic and social expectations, and heightened emotional reactivity. Such developmental dynamics may increase vulnerability to internalizing symptoms, which is consistent with the pattern observed in the present study.

The observed socioeconomic differentiation indicates that children from families with less favourable perceived financial conditions were more likely to receive lower parental evaluations of mental health and to exhibit higher levels of mood-related symptom indicators. This pattern is consistent with research linking socioeconomic disadvantage with increased exposure to psychosocial stressors and reduced access to supportive resources.

It should also be noted that the symptom indicators used in the present study may capture subclinical manifestations of emotional difficulties rather than formally diagnosed mood disorders. Parent-reported assessments can identify early warning signs or mild symptom clusters that may not meet diagnostic thresholds but still reflect meaningful variations in psychological well-being.

These findings may be interpreted within contemporary models of child and adolescent mental health, including ecological frameworks of development and social determinants of health approaches. Such perspectives emphasize that mental health outcomes emerge from interactions between individual developmental processes and broader social contexts, including family resources, educational environments, and community conditions.

The findings concerning perceived accessibility of mental health services further reinforce the systemic relevance of the study. The WHO World Mental Health Report highlights persistent global deficiencies in child and adolescent mental health service provision, including workforce shortages, long waiting times, and inequitable access [1]. Similarly, OECD analyses emphasize that unmet mental health needs among young populations remain widespread even within high-income healthcare systems [9]. The reliance on privately financed services observed in the present study is consistent with broader structural challenges documented internationally.

Several interpretive considerations should be acknowledged. Parental evaluations represent indirect assessments and may be influenced by reporting biases, health literacy, and sociocultural factors. The cross-sectional design precludes causal inference; however, the observed associations exhibit strong theoretical coherence and convergence with established empirical findings. Furthermore, the composite mood disorder risk index reflects perceived symptom frequency rather than clinical diagnosis.

Despite these limitations, the results demonstrate striking consistency with contemporary developmental and socioeconomic models of child and adolescent mental health. Age-related gradients, socioeconomic differentiation, and the high prevalence of subclinical symptomatology collectively underscore the dynamic and structurally conditioned nature of youth mental health. These findings support the need for developmentally sensitive preventive strategies, early detection mechanisms, and systemic improvements in service accessibility.

### Strengths and Limitations

The principal strength of the study lies in the large sample size, which enhances statistical power and supports stable estimation of population-level patterns. The inclusion of a broad developmental age range (6–18 years) enabled analysis of age-related gradients, while the integration of multiple sociodemographic variables allowed for a multidimensional assessment of determinants of perceived mental health. An additional advantage is the application of symptom-oriented indicators conceptually grounded in established diagnostic frameworks, facilitating a more granular evaluation beyond global subjective assessments. The use of nonparametric statistical procedures appropriate for ordinal data further strengthens the methodological rigor.

Several limitations should be acknowledged. First, the cross-sectional design precludes causal inference and limits conclusions to associations rather than directional effects. Second, reliance on parental reports introduces potential reporting biases, including perceptual distortions and limited observability of internalizing symptoms. Third, the composite mood disorder risk index reflects perceived symptom frequency rather than clinically validated diagnoses. Finally, the sample structure, characterized by a predominance of highly educated respondents, may constrain generalizability and introduce selection effects.

## 5. Conclusions

The findings of the present study provide consistent empirical support for the developmental and socioeconomic differentiation of perceived mental health status among children and adolescents. Although the majority of parents evaluated both the physical and mental health of their children as favorable, a systematic discrepancy between somatic and psychological domains was observed. Mental health was rated less positively and exhibited greater variability, suggesting that psychological functioning constitutes a more sensitive indicator of perceived well-being within this population.

A robust age-related gradient emerged across analyses. Both subjective mental health evaluations and the composite mood disorder risk index demonstrated statistically significant deterioration with increasing age. Adolescents were characterized by lower frequencies of positive mental health ratings and elevated indicators of psychological distress. This pattern aligns with established developmental psychopathology frameworks, which emphasize heightened vulnerability during adolescence due to neurobiological maturation, psychosocial transitions, and increased exposure to stressors. The results indicate that mental health difficulties should not be conceptualized as uniformly distributed across childhood but rather as dynamically shaped by developmental stage.

Socioeconomic factors, particularly family financial situation, exhibited the strongest and most consistent associations with mental health indicators. Children from economically disadvantaged families demonstrated markedly less favorable mental health evaluations and substantially higher mood disorder risk. The magnitude of these differences suggests that material conditions operate not merely as background variables but as critical determinants of psychological functioning. These findings are congruent with stress-exposure and social determinants of health models, which posit that chronic financial strain contributes to cumulative psychosocial burden, diminished access to resources, and elevated risk of emotional dysregulation.

Parental occupational activity also demonstrated measurable associations with child mental health outcomes, albeit with smaller effect sizes. The observed pattern suggests that employment-related stability may exert a protective function, potentially mediated through reduced economic uncertainty, structured family environments, and enhanced psychosocial security. In contrast, parental educational attainment did not significantly differentiate mental health assessments, indicating that educational capital alone may not sufficiently buffer against mental health risks in the absence of broader socioeconomic resources.

The prevalence of symptom-related indicators points toward a substantial proportion of children experiencing subclinical psychological difficulties. Elevated rates of concentration problems, affective distress, negative self-referential cognitions, and somatic correlates of psychological strain (sleep disturbances, fatigue) suggest that psychological burden is widespread, even among children globally evaluated as mentally healthy. This discrepancy underscores the importance of moving beyond global subjective assessments toward more granular symptom-based screening approaches.

From a public health perspective, the findings highlight the need for developmentally targeted and socioeconomically sensitive mental health strategies. The clear age-related increase in psychological risk indicators suggests that preventive interventions should intensify during late childhood and adolescence. Simultaneously, the pronounced socioeconomic gradient implies that mental health policies must incorporate structural considerations, including economic inequality and accessibility of services.

The widespread perception of insufficient availability of publicly funded psychiatric and psychological care, coupled with high reliance on privately financed services, indicates the presence of systemic barriers within the mental health care infrastructure. These structural constraints may contribute to delayed intervention, unequal access to treatment, and the amplification of health disparities.

Collectively, the results support several overarching conclusions. First, mental health constitutes a domain of increased vulnerability relative to physical health within the pediatric population. Second, developmental stage represents a primary axis of differentiation in psychological risk. Third, socioeconomic adversity, particularly financial disadvantage, functions as a central risk factor for compromised mental health. Finally, the patterns observed necessitate an integrated approach combining early detection, preventive programming, and systemic improvements in service accessibility.

The study reinforces the conceptualization of child and adolescent mental health as a product of interacting developmental and environmental influences, requiring coordinated responses at the clinical, educational, and policy levels.

## Figures and Tables

**Table 1:** Overall parental evaluation of children’s physical and mental health.

Health Status	Physical Health n (%)	Mental Health n (%)	χ^2^	*p*-Value	Effect Size (Cramér’s V)
Very poor	17 (1.4)	8 (0.7)	214.63	<0.001	0.30
Poor	15 (1.3)	54 (4.6)			
Fair	123 (10.5)	259 (22.0)			
Good	587 (49.9)	607 (51.6)			
Very good	435 (37.0)	249 (21.2)			
Total	1177 (100.0)	1177 (100.0)			

**Table 2:** Distribution of responses to mood disorder risk indicators.

Item	Never %	Rarely %	Often %	Very Often %	Always %	Total %	Clinically Relevant Symptoms * %
How often did your child appear sad or depressed?	2.9	68.1	24.6	4.2	0.2	100.0	29.0
How often did your child have sleep problems (e.g., difficulty falling asleep or waking at night)?	20.7	55.7	17.3	5.1	1.1	100.0	23.5
How often did your child have difficulties with concentration or attention?	4.2	43.3	35.3	14.5	2.7	100.0	52.5
How often did your child express negative thoughts about themselves (e.g., feeling worthless or incapable)?	24.5	52.7	16.8	5.4	0.6	100.0	22.8
How often did your child show signs of withdrawal from family or friends?	31.9	47.9	14.4	5.4	0.3	100.0	20.1
How often did your child appear tired or lacking energy for most of the day?	15.0	56.2	20.7	7.5	0.5	100.0	28.7
How often did your child display negative emotions such as sadness, despair, or anxiety?	16.6	60.4	18.2	4.7	0.2	100.0	23.1

* Clinically relevant symptoms = Often + Very often + Always.

**Table 3:** Prevalence of clinically relevant internalizing symptom domains.

Symptom Domain	Prevalence of Clinically Relevant Symptoms (%)
Affective symptoms (sad/depressed mood)	29.0
Sleep disturbances	23.5
Cognitive difficulties (concentration/attention)	52.5
Negative self-referential cognitions	22.8
Social withdrawal	20.1
Fatigue/low energy	28.7
Negative emotional states	23.1

**Table 4:** Mood disorder risk index (scale 1–5) across selected variables.

Variable	Min	Max	Mean	SD	Test	*p*-Value	Effect Size
Age group					H = 62.74	<0.001	η^2^ (H) = 0.05
6–9 years	2.29	5.00	3.95	0.47			
10–14 years	1.57	5.00	3.78	0.57			
15–18 years	1.43	5.00	3.65	0.67			
Parental education					H = 3.12	0.210	
Higher education	1.57	5.00	3.79	0.57			
Secondary education	1.43	4.86	3.80	0.63			
Below secondary	2.00	4.71	3.89	0.70			
Parental occupational status					U = 159,284	0.084	r = 0.05
Economically active	1.43	5.00	3.80	0.58			
Other status	1.57	5.00	3.77	0.69			
Family financial situation					H = 71.39	<0.001	η^2^ (H) = 0.06
Poor/very poor	1.43	4.43	3.25	0.94			
Average	2.00	5.00	3.77	0.60			
Good/very good	1.86	5.00	3.83	0.55			

## Data Availability

The raw data supporting the conclusions of this article will be made available by the authors on request.
